# Increased risk of cardiovascular and renal disease, and diabetes for all women diagnosed with gestational diabetes mellitus in New Zealand—A national retrospective cohort study

**DOI:** 10.1111/1753-0407.13535

**Published:** 2024-04-10

**Authors:** Barbara M. Daly, Zhenqiang Wu, Krishnarajah Nirantharakumar, Lynne Chepulis, Janet A. Rowan, Robert K. R. Scragg

**Affiliations:** ^1^ Faculty of Medical and Health Sciences University of Auckland Auckland New Zealand; ^2^ Department of Geriatric Medicine University of Auckland Auckland New Zealand; ^3^ Professor in Health Data Science and Public Health, Institute of Applied Health Research University of Birmingham Birmingham UK; ^4^ School of Health University of Waikato Hamilton Waikato New Zealand; ^5^ National Women Health at Auckland City Hospital Auckland New Zealand; ^6^ School of Population Health University of Auckland Auckland New Zealand

**Keywords:** cardiovascular disease, dyslipidemia, gestational diabetes, hypertension, renal disease, type 2 diabetes

## Abstract

**Background:**

Gestational diabetes mellitus increases the risk of developing type 2 diabetes. The aim of this study is to compare cardiometabolic and renal outcomes for all women in New Zealand with gestational diabetes (2001–2010) with women without diabetes, 10–20 years following delivery.

**Methods:**

A retrospective cohort study, utilizing a national dataset providing information for all women who gave birth between 1 January 2001 and 31 December 2010 (*n* = 604 398). Adolescent girls <15 years, women ≥50 years and women with prepregnancy diabetes were excluded. In total 11 459 women were diagnosed with gestational diabetes and 11 447 were matched (for age and year of delivery) with 57 235 unexposed (control) women. A national hospital dataset was used to compare primary outcomes until 31 May 2021.

**Results:**

After controlling for ethnicity, women with gestational diabetes were significantly more likely than control women to develop diabetes—adjusted hazard ratio (HR) 20.06 and 95% confidence interval (CI) 18.46–21.79; a first cardiovascular event 2.19 (1.86–2.58); renal disease 6.34 (5.35–7.51) and all‐cause mortality 1.55 (1.31–1.83), all *p* values <.0001. The HR and 95% CI remained similar after controlling for significant covariates: diabetes 18.89 (17.36–20.56), cardiovascular events 1.79 (1.52–2.12), renal disease 5.42 (4.55–6.45), and all‐cause mortality 1.44 (1.21–1.70). When time‐dependent diabetes was added to the model, significance remained for cardiovascular events 1.33 (1.10–1.61), *p* = .003 and renal disease 2.33 (1.88–2.88), *p* < .0001 but not all‐cause mortality.

**Conclusions:**

Women diagnosed with gestational diabetes have an increased risk of adverse cardiometabolic and renal outcomes. Findings highlight the importance of follow‐up screening for diabetes, cardiovascular risk factors, and renal disease.

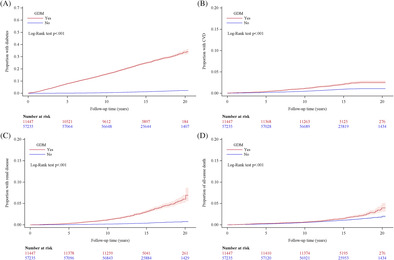

## Graphical abstract

Highlights

- Women diagnosed with gestational diabetes were significantly more likely to develop renal disease, have a major cardiovascular event, and develop hypertension and dyslipidemia following delivery compared with women without diabetes in pregnancy.
- The risk of developing type 2 diabetes for these women remains 20 times higher than for women without diabetes in pregnancy, despite knowledge of this association for over 50 years.
- European women diagnosed with gestational diabetes have the highest risk of developing type 2 diabetes compared with Māori, Pacific, and Asian women.

## 1 BACKGROUND

The prevalence of gestational diabetes mellitus (GDM) in New Zealand, based on regional reports, has increased from 1.3% in 2001^1^ to 6.2% in 2019^2^ largely due to the obesity epidemic^1^ and changes in population demographics.^3^ Each year 3000 to 4000 women in New Zealand are diagnosed with GDM.^1^ Compared with European/other women, whose prevalence of GDM is 6%,^4^ the prevalence is significantly higher for Indian (28%), Asian (17%), Pacific (19%), and Middle Eastern, Latin American, and African (14%) women.^5^, ^6^ It is also increased for Māori women (10%), which is an underestimate, largely due to a lack of screening.^5^, ^6^

Women with GDM typically develop elevated glucose levels during the second trimester when women are unable to compensate for the increase in insulin resistance in the second half of pregnancy.^7^ In addition to the increased risk for maternal and neonatal complications during pregnancy and delivery,^1^, ^8^ these women have increased risks of subsequent beta cell dysfunction^9^, ^10^ and insulin resistance^11^ and adverse cardiometabolic outcomes in the long term.

Compared with control women, there is a 10–20 fold increase in the incidence rate for developing type 2 diabetes in women with GDM.^8^, ^12^ Twelve large population studies of mostly European women show an increased risk for women with GDM developing cardiovascular disease (CVD) from Canada,^13^, ^14^, ^15^ the United States,^16^, ^17^, ^18^, ^19^ Europe,^20^, ^21^, ^22^, ^23^ the United Kingdom,^24^ and Israel.^25^ A small number of studies have reported a positive association between GDM and renal disease,^26^, ^27^, ^28^, ^29^ and among women who also developed type 2 diabetes.^13^, ^30^, ^31^ Although the relationship between GDM and renal disease is not well understood, genetic studies show several loci are associated with down regulation of renal nuclear factor erythroid 2‐related factor 2 and reduced glomerular filtration.^31^ Animal modeling also suggests that increasing advanced glycation end products bind to and reduce tubule cell function, as they filtrate through the nephrons.^32^ A meta‐regression pooled analysis reported increased risks for women with GDM of developing hypertension and dyslipidemia and a risk ratio of 1.98 (95% confidence interval [CI] 1.57–2.50) for major cardiovascular events,^33^ 1.98 (1.57–2.50) for fatal and nonfatal myocardial infarction, and 1.25 (1.07–1.48) for stroke,^34^ and at an earlier age, compared with women who were not diagnosed with GDM during pregnancy.^33^, ^34^

The aim of this study is to identify all women, in a multiethnic population, diagnosed with GDM in New Zealand between 1 January 2001, and 31 December 2010, and compare cardiometabolic (diabetes, cardiovascular, mortality, hypertension, and dyslipidemia) and renal outcomes in the 10 to 20 years following delivery with women who were not diagnosed with any diabetes before or during pregnancy (matched for age and year of delivery).

## 2 METHODS

**FIGURE 1:**
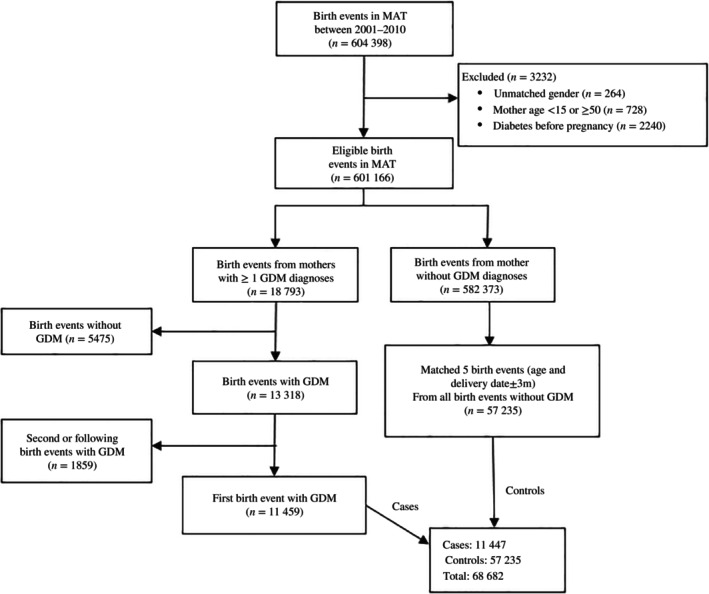
Total birth events in New Zealand between 2001 and 2010, 11 459 women diagnosed with gestational diabetes mellitus and 11 447 matched with control women (*n* = 57 235). GDM, gestational diabetes mellitus; MAT, National Maternity Collection.

A retrospective cohort study was carried out utilizing the National Maternity Collection (MAT) dataset that included all births in New Zealand between 1 January 2001 and 31 December 2010 (*n* = 604 398). MAT data are collected from primary maternity services, lead maternity carer claims for payment, and hospital records using the National Minimum Dataset (NMDS) for inpatients and day‐patients during pregnancy, birth, and the postnatal period. Adolescent girls <15 years, women ≥50 years, women whose gender was incorrectly recorded and women with type 1 or type 2 diabetes prior to pregnancy were excluded from this study (Figure 1).

### 2.1 Study population

During the study period, an early pregnancy 75 g oral glucose tolerance test (OGTT) was recommended for women identified with clinical risk factors for undiagnosed diabetes or impaired glucose tolerance. Women with a fasting glucose ≥5.5 mmol/L and/or a 2‐h glucose ≥9.0 mmol/L were referred to a maternity diabetes service. During the study period (prior to 2011), these women were labeled as having GDM. Women with undiagnosed diabetes were reclassified following a 75 g OGTT at 6–8 weeks postdelivery.^1^, ^35^ Screening for GDM was recommended at 24–28 weeks for women who had not had a test or had had a nondiagnostic test in early pregnancy. Women without recognized risk factors for GDM were asked to perform a screening test with a nonfasting 50 g glycose challenge (then called a polycose test), and if the 1‐h result was 7.8–11.0 mmol/L, they were asked to undertake a diagnostic 75 g OGTT.^1^, ^35^ Women with a polycose result >11.0 mmol/L were typically referred to the maternity diabetes clinic without performing a further test. The recommendation for women with clinical risk factors, who were not diagnosed with GDM in early pregnancy, was a repeat 75 g OGTT between 24 and 28 weeks gestation. Other women were screened with a nonfasting 50 g glucose load. If the 1‐h glucose result was ≥7.8 mmol/L, a diagnostic 75 g OGTT was recommended. The glucose thresholds for a diagnosis of GDM were the same as in early pregnancy.^1^, ^35^

Women diagnosed with GDM during at least one pregnancy (exposed cohort) were included in this study and had 18 793 birth events. After excluding duplicate mothers, who had more than one pregnancy‐related GDM, a total of 11 459 women were diagnosed with GDM. Of those, 11 447 (99.9%) were randomly matched in a ratio 1:5 (for age and year of delivery +/− 3 months) using information associated with their first GDM pregnancy and compared with five unexposed or control women (*n* = 57 235) who were not identified with any diabetes during pregnancy. Twelve women diagnosed with GDM, at the extreme age ranges, were not able to be matched with control women.

Women were identified and excluded from this study if they had existing type 1 or 2 or any other diabetes‐related hospitalization (except if diagnosed with GDM) prior to delivery using a previously developed method for constructing the NZ Virtual Diabetes Register (VDR)^36^, ^37^ and the NMDS for hospital admissions. Essentially, this method utilizes the national Pharmaceutical Information Database (PHARMS) to identify women with established type 1 or 2 diabetes by dispensed medications for hyperglycemia (oral hyperglycemics or insulin) at any time before pregnancy, during early pregnancy, and throughout pregnancy and up to 2 weeks after delivery.

The MAT dataset was utilized to describe and compare biographical details for women with GDM with control women (Table 1). Middle Eastern, Latin American, Hispanic, and African women, and women who did not state their ethnicity, accounted for 1.9% of the total cohort and were combined with European women for all analyses. Ethical approval was not required for this report, as national anonymized datasets were utilized and individuals are not able to be identified—confirmed by the NZ Health and Disability Ethics committee (20 January 2023).

**TABLE 1:** Baseline characteristics for women diagnosed with gestational diabetes mellitus and control women.

Characteristic	Women with GDM (*N* = 11 447)	Control Women (*N* = 57 235)	*p* value
Age (years)			1.00
Mean (SD)	32.3 (5.7)	32.3 (5.7)	
Median (IQR)	33.0 (28.0–36.0)	33.0 (28.0–36.0)	
Ethnicity, *n* (%)			<.0001
European/other^a^	4757 (41.6)	36 431 (63.7)	
Māori	1747 (15.3)	10 030 (17.5)	
Pacific	2200 (19.2)	5123 (9.0)	
Asian	2743 (24.0)	5651 (9.9)	
Deprivation decile			<.0001
Mean (SD)	6.5 (2.8)	5.8 (2.9)	
Median (IQR)	7.0 (4.0–9.0)	6.0 (3.0–8.0)	
Missing values	20	375	
Deprivation decile^b^, *n* (%)			<.0001
Least deprived area	2099 (18.4)	14 760 (26.0)	
Medium deprived area	4149 (36.3)	21 794 (38.3)	
Most deprived area	5179 (45.3)	20 306 (35.7)	
Missing value	20	375	
Deprivation status^c^, *n* (%)			<.0001
Highest	1384 (12.1)	9856 (17.3)	
High	1611 (14.1)	10 419 (18.3)	
Middle	2160 (18.9)	11 077 (19.5)	
Low	2587 (22.6)	11 751 (20.7)	
Lowest	3685 (32.3)	13 757 (24.2)	
Missing values	20	375	
Urban/rural status, *n* (%)			<.0001
City	8592 (78.2)	37 446 (67.9)	
Semirural	707 (6.4)	4918 (8.9)	
Rural	1371 (12.5)	10 012 (18.2)	
Remote	319 (2.9)	2743 (5.0)	
Missing values	458	2116	
Regional Health Authorities (RHA), *n* (%)			<.0001
Northern	6093 (53.3)	23 262 (40.7)	
Midland	1874 (16.4)	10 323 (18.1)	
Central	1565 (13.7)	11 422 (20.0)	
Southern	1904 (16.6)	12 081 (21.2)	
Missing values/overseas	11	147	
Resident status, *n* (%)			<.0001
Yes	10 558 (92.2)	53 476 (94.1)	
No	887 (7.8)	3369 (5.9)	
Missing values	2	390	
Registered with lead maternity carer, *n* (%)			<.0001
Yes	10 618 (92.8)	54 263 (94.8)	
No	829 (7.2)	2972 (5.2)	
No. of previous births			<.0001
Nil	4023 (40.3)	19 032 (36.7)	
1	2870 (28.8)	16 703 (32.2)	
2–3	2147 (21.5)	12 274 (23.7)	
4–6	809 (8.1)	3287 (6.3)	
>7	124 (1.2)	515 (0.9)	
Missing values	1474	5424	
Preeclampsia, *n* (%)			<.0001
Yes	1347 (11.8)	2848 (5.0)	
No	10 100 (88.2)	54 387 (95.0)	
Smoking^d^, *n* (%)			<.0001
Yes	316 (2.8)	2460 (4.3)	
Unknown	7504 (65.6)	34 276 (59.9)	
No	3627 (31.7)	20 499 (35.8)	
Cardiovascular medications 1‐year prior to delivery^e^, *n* (%)			<.0001
Yes	547 (4.8)	1021 (1.8)	
No	10 900 (95.2)	56 214 (98.2)	
Delivery and infant characteristics			
Gestational age (week)			<.0001
Mean (SD)	38.1 (1.9)	39.1 (2.0)	
Median (IQR)	38.0 (38.0–39.0)	40.0 (38.0–40.0)	
Missing values	294	2117	
Preterm, *n* (%)			<.0001
Yes	1318 (11.8)	3698 (6.7)	
No	9835 (88.2)	51 420 (93.3)	
Missing values	294	2117	

Abbreviation: IQR, interquartile range.

^a^ Middle Eastern, Latin American, Hispanic, and African women, and women who did not state their ethnicity, accounted for 1.9% of the total cohort and were combined with European women for all analyses.

^b^ Deprivation decile (low 1–3, medium 4–7, high 8–10).

^c^ Deprivation status (highest 1–2, high 3–4, middle 5–6, low 7–8, and lowest 9–10).

^d^ Smoking at two weeks post‐delivery with improved records from 2008 to 2009.

^e^ Cardiovascular medications (antihypertensives, diuretics, anticoagulants, cardiac, nitrates and lipid lowering).

### 2.2 Follow‐up data sets

For outcomes, women diagnosed with GDM were compared with control women who were not diagnosed with any diabetes throughout their pregnancy. For all primary outcomes, the MAT dataset was linked with the latest available data from the NMDS for hospital admissions, treatment lasting for at least 3 hours or death. Coding for renal, cardiovascular, and mortality outcomes were based on the modified Australian Coding Standards for the International Classification of Diseases (ICD) for cardiovascular events (ICD, Tenth Revision, Australian Modification [ICD‐10‐AM])—outlined by Wu et al^38^ (Supplementary File 1), any diabetes (E10 [including type 1] E11 E13 E14 O240 O241 O242 O243), acute and chronic kidney disease (N00‐N99 N17‐N19), and for death events (ICD‐10‐AM). Secondary outcomes were the development of hypertension or dyslipidemia, based on the PHARMS dataset of two or more related medications (antihypertensive, lipid modifying agents) dispensed at any time at least 2 weeks following delivery. PHARMS was also utilized for dispensed medications using encrypted National Health Index numbers to identify women dispensed two or more other cardiovascular‐related medications (diuretics, anticoagulants, antiarrhythmic, calcium channel modifiers, nitrates) after delivery to identify potential confounding covariables. For cardiovascular events, women who had any cardiovascular‐related medications dispensed were controlled for in the fully adjusted models. When calculating adjusted hazard ratios (HRs) for women with GDM who developed hypertension and dyslipidemia (utilizing dispensed hypertensive or lipid modifying medications following delivery), women were excluded from the analyses who had hypertensive or lipid modifying medications dispensed 1 year prior to delivery, respectively.

### 2.3 Statistical Analyses

**FIGURE 2:**
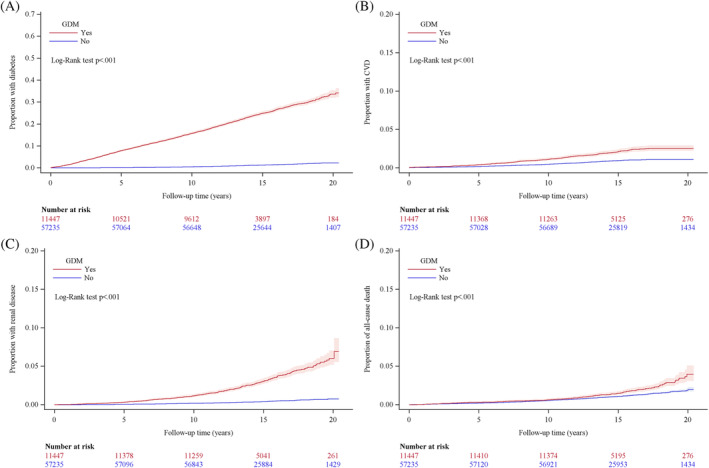
Cumulative proportion of women diagnosed with gestational diabetes mellites and control women who developed diabetes (A), had cardiovascular event (B), renal disease (C), or all‐cause mortality (D). GDM, gestational diabetes mellitus.

**FIGURE 3:**
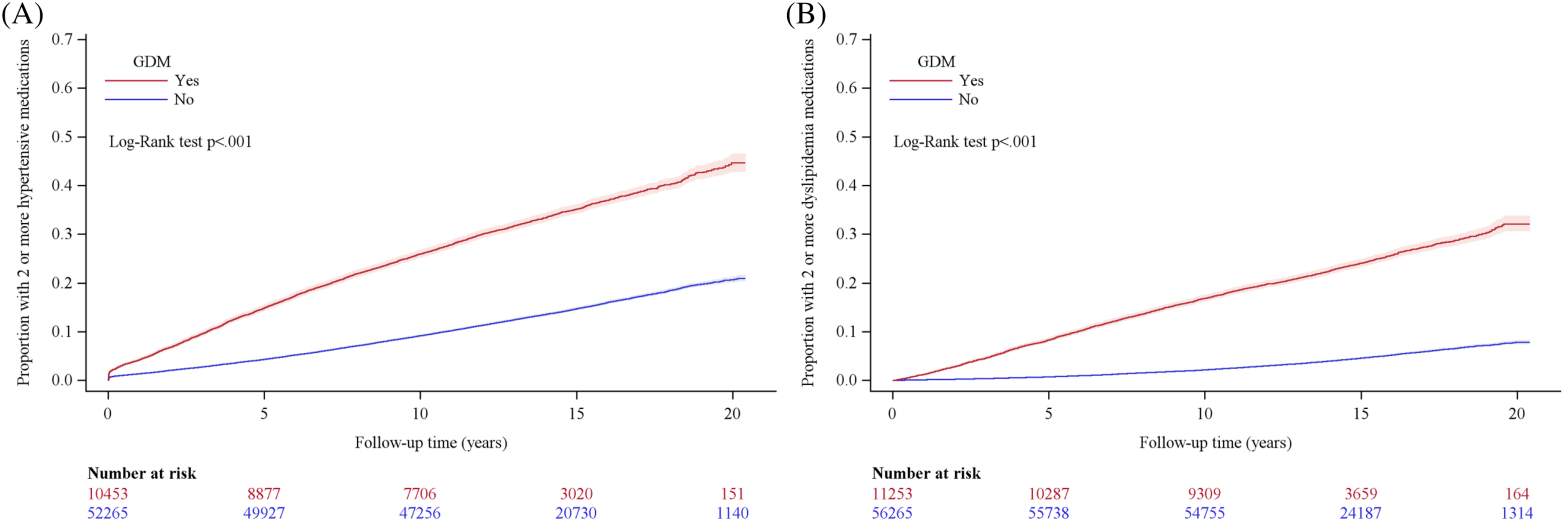
Cumulative proportion of women diagnosed with GDM and control women dispensed two or more hypertensive medications (A) or two or more lipid modifying medications (B). GDM, gestational diabetes mellitus.

**FIGURE A1:**
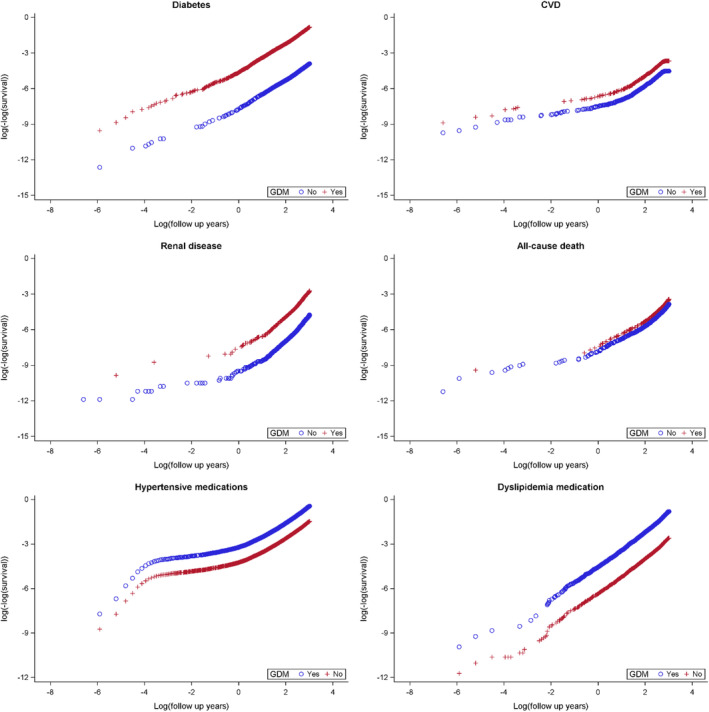
Exploring the Proportional Hazard Assumption—log(−log [survival]) against log(survival time) for each outcome.

SAS version 9.4 (SAS Institute, Cary, NC, 2013) was used for all statistical calculations. PROC FREQ was used to generate frequency and percentage for categorical variables. PROC MEANS was used to generate mean and SD for continuous variables. Chi square or *t* tests were used to compare women during their first GDM pregnancy with control women for all categorical and continuous baseline characteristics. PROC PHREG was utilized to compute Cox proportional HR and 95% CI for time‐to‐first event for the six outcomes for women with GDM and control women between 2001 and 2021. Time‐dependent diabetes was included in the fully adjusted Cox model for other outcomes. The proportional hazards assumption of the Cox models was assessed using the graphical approach (Kaplan–Meier curves and Log–Log Survival curves) and indicate that this was not violated by exposed and unexposed women (Figures 2 and 3 and Appendix Figure A1). PROC LIFETEST was used to calculate Kaplan–Meier cumulative incidences for each cardiometabolic outcome.

## 3 RESULTS

Figure 1 outlines the number of total maternal birth events (*N* = 604 398) in New Zealand between 1 January 2001, and 31 December 2010. Women excluded from the study (*n* = 3232) included those under 15 or ≥50 years of age, incorrect gender records and those identified with type 1 or 2 diabetes (utilizing dispensed glycemic medications) prior to pregnancy. Of the total birth events, 18 793 were identified with mothers diagnosed with GDM. After excluding duplicate mothers, 11 459 women were included in the study and 11 447 were able to be matched with control women, who were not diagnosed with GDM during their pregnancy.

Women with GDM compared with control women were significantly more likely to be Asian or Pacific women, economically deprived and reside in urban areas (Table 1) and particularly in central (18%) and South (19%) Auckland compared with 12% of control women—data not shown. Women were also more likely to be diagnosed with GDM if they were nonresidents, not registered with a lead maternal carer, experienced a first or ≥4 previous births, or develop preeclampsia compared with control women (Table 1). Women with GDM were also more likely to have been dispensed at least one cardiovascular‐related medication within the 12 months prior to delivery and have a shorter gestation at delivery compared with control women. Infants of mothers who were diagnosed with GDM were more likely to be premature at birth than infants of control mothers (Table 1).

Table 2 confirms the lifetime risk for women diagnosed with GDM developing diabetes after controlling for ethnicity, in addition to matching for age and delivery year, HR (95% CI): 20.06 (18.46–21.79); (Model 1). Using the same model, women with GDM had over double the risk of being admitted to hospital with a first cardiovascular event 2.19 (1.86–2.58), over six times more likely to be admitted for renal disease 6.34 (5.35–7.51), an increased risk of premature death 1.55 (1.31–1.83), and increased risks of being dispensed at least two antihypertensive 2.77 (2.66, 2.88) or lipid modifying 5.61 (5.31–5.94) medications after delivery. The HR (95% CI) were similar for each outcome after controlling for all significant covariates maternal deprivation status, Regional Health Authority region, resident status, unregistered with a lead maternity carer, number of previous births, preeclampsia, smoking, cardiovascular medications dispensed 1 year prior to delivery, and premature birth (Model 2). The HR (95% CI) were: diabetes 18.89 (17.36–20.56); cardiovascular events 1.79 (1.52–2.12); renal disease 5.42 (4.55–6.45), and all‐cause mortality 1.44 (1.21–1.70). The effect of high infant birthweight (>5000 g), using (Model 2) had very little effect on HR for all primary outcomes.

**TABLE 2:** Adjusted hazard ratios (HR) for adverse events following delivery in women identified with gestational diabetes mellitus (*n* = 11 447) compared with control women (matched for age and time of delivery, *n* = 57 235).

Outcome variables	Women with GDM, *n* (%)	Control women, *n* (%)	Model 1^a^		Model 2^b^		Model 3^c^	
			HR (95%CI)	*p* value	HR (95%CI)	*p* value	HR (95%CI)	*p* value
Diabetes	2769 (24.2)	738 (1.29)	20.06 (18.46–21.79)	<.0001	18.89 (17.36–20.56)	<.0001	NA	NA
Outcomes based on hospitalization or deaths								
Total CV events	228 (2.0)	492 (0.9)	2.19 (1.86–2.58)	<.0001	1.79 (1.52–2.12)	<.0001	1.33 (1.10–1.61)	.003
Renal disease	358 (3.1)	238 (0.4)	6.34 (5.35–7.51)	<.0001	5.42 (4.55–6.45)	<.0001	2.33 (1.88–2.88)	<.0001
All‐cause mortality	191 (1.7)	629 (1.1)	1.55 (1.31–1.83)	<.0001	1.44 (1.21–1.70)	<.0001	0.82 (0.67–1.01)	.06
Outcomes based on 2 or more dispensings of medications after delivery								
Hypertensive medications	3626 (34.7)	7693 (14.7)	2.77 (2.66–2.88)	<.0001	2.47 (2.37–2.58)^d^	<.0001	1.70 (1.62–1.78)	<.0001
Dyslipidemia medications	2658 (23.6)	2597 (4.6)	5.61 (5.31–5.94)	<.0001	5.13 (4.87–5.46)^e^	<.0001	2.84 (2.66–3.04)	<.0001

Abbreviations: 95% CI, 95% confidence interval; CV, cardiovascular; GDM, gestational diabetes mellitus; HR, hazard ratio; NA, not appliable.

^a^ Model 1 adjusted for ethnicity in addition to matching for age and delivery period.

^b^ Model 2 adjusted for the variables in Model 1 plus maternal deprivation status (highest 1–2, high 3–4, middle 5–6, low 7–8, lowest 9–10); Regional Health Authority (Northern, Midland, Central, and Southern), tesident status, unregistered with lead maternity carer, number of previous births (nil, 1, 2–3, 4–6, ≥7), Preeclampsia, smoking (yes, unknown, and no), cardiovascular medications 1 year prior to delivery, and preterm birth.

^c^ Model 3 adjusted for all variables in Model 2 plus time‐dependent type 2 diabetes.

^d^ Same variables in Model 2, excluding women who were dispensed hypertensive medications 1 year prior to delivery.

^e^ Same variables in Model 2, excluding women who were dispensed lipid modifying medications 1 year prior to delivery.

However, when time‐dependent development of diabetes was added to the model (Model 3—all variables in model 2 and time‐dependent diabetes), HRs were greatly reduced compared to Model 2, indicating that about half of the increased risk for these outcomes was related to a diagnosis of diabetes after pregnancy. The HR for all outcomes remained significant (*p* < .05) except for all‐cause mortality (*p* = .06).

Figure 2 outlines the cumulative proportions of women diagnosed with GDM who develop diabetes, are hospitalized with a first cardiovascular event and/or renal disease and all‐cause mortality were all significantly higher compared with control women after matching for age and year of birth. A linear increased risk with time since delivery was found for all outcomes throughout the 20‐year follow‐up period. However, the risk of a first cardiovascular event plateaued after an average 15‐year of follow‐up‐period and the increased risk for all‐cause mortality occurred from 12 years postdelivery. The risk was almost exponential for renal disease from about 14 years postdelivery.

Figure 3 shows the cumulative proportion of women with GDM compared with control women who were dispensed hypertensive and lipid modifying medications over 20 years following delivery. A similarly large increased risk was found for developing hypertension and dyslipidemia for women diagnosed with GDM, and this continued to increase throughout the 20‐year follow‐up period, compared with control women.

Table 3 reports the baseline characteristics (ethnicity, socio‐economic status, number of previous births and preeclampsia) that significantly modify the association between GDM and developing diabetes following delivery, after controlling for all variables in the adjusted model (*p* value for interaction <.05). In the subgroup analyses by baseline characteristics, European/other women with GDM had the highest hazard of developing diabetes following delivery (HR = 37.45, 95% CI = 31.79–44.12), followed by Māori women 16.44 (14.16–19.09) compared with their respective controls. Less economically deprived women with GDM (HR = 32.65, 95% CI = 23.40–45.54), primipara women with GDM (HR = 31.64, 95% CI = 25.93–38.61) and women with GDM who did not develop preeclampsia were more likely to develop diabetes (HR = 19.92, 95% CI = 18.19–21.81) than women who also developed preeclampsia (HR = 12.48, 95% CI =9.87–15.78) compared with their respective controls. Subgroup analyses for other primary outcomes (renal disease, CVD, and mortality) also showed similar adjusted HR. Although *p* values for assessing interaction were close to .05, indicting significance is likely to be a chance finding. The latter subgroup analyses have not been included due to small numbers.

**TABLE 3:** Adjusted hazard ratios (HR) for women with gestational diabetes (*n* = 11 447) developing diabetes postdelivery compared with control/unexposed women (*n* = 57 235).

Variables	Women with GDM (*N* = 11 447) *N* (%)	Control women (*N* = 57 235) *N* (%)	Adjusted HR^a^ (95% CI)	*p* value	*p* value for interaction test^b^
Total diabetes	2769 (24.2)	738 (1.29)	18.89 (17.36–20.56)	<.0001	
Ethnicity					<.0001
European/other	850 (17.9)	178 (0.5)	37.45 (31.79–44.12)	<.0001	
Māori	620 (35.5)	256 (2.6)	16.44 (14.16–19.09)	<.0001	
Pacific	980 (44.6)	254 (5.0)	11.15 (9.67–12.86)	<.0001	
Asian	319 (11.6)	50 (0.9)	13.34 (9.78–18.19)	<.0001	
Socioeconomic status^c^					.024
Highest	203 (14.7)	45 (0.5)	32.65 (23.40–45.54)	<.0001	
High	275 (17.1)	68 (0.7)	26.83 (20.41–35.28)	<.0001	
Middle	421 (19.5)	73 (0.7)	33.71 (26.15–43.47)	<.0001	
Low	633 (24.5)	168 (1.4)	18.69 (15.69–22.28)	<.0001	
Lowest	1229 (33.4)	368 (2.7)	13.58 (12.03–15.33)	<.0001	
Previous births^d^					<.0001
Nil	775 (19.3)	116 (0.6)	31.64 (25.93–38.61)	<.0001	
1	574 (20.0)	139 (0.8)	23.93 (19.77–28.96)	<.0001	
2–3	633 (29.5)	222 (1.8)	15.48 (13.20–18.17)	<.0001	
4–6	344 (42.5)	137 (4.2)	11.01 (8.93–13.56)	<.0001	
≥7	49 (39.5)	25 (4.9)	9.86 (5.73–16.99)	<.0001	
Preeclampsia					<.0001
Yes	460 (34.2)	87 (3.1)	12.48 (9.87–15.78)	<.0001	
No	2309 (22.9)	651 (1.2)	19.92 (18.19–21.81)	<.0001	

Abbreviations: 95% CI, 95% confidence interval; GDM, gestational diabetes mellitus; HR, hazard ratio.

^a^ Adjusted for age, year of delivery, ethnicity, maternal deprivation status (highest 1–2, high 3–4, middle 5–6, low 7–8 and lowest 9–10); Regional Health Authority (Northern, Midland, Central, and Southern), resident status, unregistered with lead maternity carer, number of previous births (nil, 1, 2–3, 4–6, and ≥7), preeclampsia, smoking (yes, unknown, and no), cardiovascular medications 1 year prior to delivery.

^b^ The effects of interactions were tested by including all interaction terms between GDM and each variable in the fully adjusted model.

^c^ NZ deprivation decile: highest (1, 2), high (3, 4), middle (5, 6), low (7, 8), and lowest (9, 10).

^d^ Parity based on self‐reported previous number of all births.

## 4 DISCUSSION

A total of 604 398 hospital birth events in New Zealand were identified between 2001 and 2010 and closely correlates with total births in New Zealand over that period.^3^ The 11 459 women diagnosed with GDM in this cohort was less than the estimated number (*n* = 15 400), based on the expected prevalence of 1.3% in 2001 and an annual increase of 13.9%.^1^ This underestimation is likely due to a lack of screening for GDM, particularly for Māori women.^5^, ^6^

Women diagnosed with GDM had a 20‐fold increase in developing diabetes, over double the risk for having a first cardiovascular event, a 6‐fold increased risk for admission to hospital for renal disease, a 1.5‐fold increased risk of a premature death, over twice the risk for developing hypertension and a 5‐fold risk of developing dyslipidemia compared with control women. The increased risk for all outcomes in women with GDM remained, after controlling for the development of diabetes following delivery but not for all‐cause mortality (Table 2). This suggests that the increased risk of adverse outcomes in GDM women is partly due to developing diabetes after their initial GDM pregnancy, but that other factors besides diabetes are also increasing the risk of adverse outcomes. The finding for cardiovascular events was similar to that separately reported for the Ontario study for women with GDM who developed type 2 diabetes HR (95% CI), 2.82 (2.41–3.30) compared with women with GDM who did not develop type 2 diabetes, 1.30 (1.07–1.59).^13^ Current results for renal disease were similar to four previous studies among American^27^ and Black American^29^ women; Israeli women, whose risk increased with each additional GDM pregnancy^26^; and aboriginal women.^28^ Similar results were reported for women with GDM who developed diabetes following delivery in Demark,^30^ Sweden,^31^ and Ontario for women requiring dialysis 7.52 (5.24–10.81) but contrasted with their finding for women who did not develop type 2 diabetes 1.25 (0.62–2.52).^13^ Findings from this NZ study also show that women who develop GDM are more likely to develop hypertension and dyslipidemia following delivery (also reported from the large Danish cohort study),^22^ both of which are associated with increasing insulin resistance, metabolic syndrome,^39^ type 2 diabetes, and an increased risk of cardiovascular events.^33^

Significant interactions were found for the risk of developing diabetes, mainly between ethnic groups (Table 3). The relevance of this is unclear and could be partly due to variation in risk among the comparison group for European/other women and the risk ratio, as the risk difference between GDM and control women is larger among Māori and Pacific women. Further studies are required to validate findings of the effects of interaction for ethnicity, socioeconomic status, number of previous births, and preeclampsia^17^ on modifying the risk of developing diabetes. Subgroup analysis showed European/other women with GDM have a higher risk of being diagnosed with developing diabetes followed by Māori and Asian women whereas Pacific women had the lowest risk compared with women without GDM (Table 3). It is known that rates of diabetes in the community are higher for Pacific, Māori, and Asian women,^40^ so this finding may reflect a lower uptake of long‐term screening for diabetes and other CV risk factors in these higher risk populations.^5^, ^41^ European women who develop GDM may also be more insulin resistant and at higher risk of developing type 2 diabetes compared with European women without GDM.

Two recent reviews reported a higher risk for non‐European women with GDM developing type 2 diabetes when comparing populations,^8^, ^12^ and one review reported a lower risk for Asian women developing metabolic syndrome.^39^ There are few studies that examine the risk of developing cardiovascular and renal disease for non‐European women with a history of GDM.

NZ best practice guidelines recommend all women who develop GDM are funded for an annual diabetes screen following delivery.^42^, ^43^ Findings from this study highlight the increased risk of cardiovascular and renal disease, and the contributing role of developing diabetes following delivery.^44^ Postpartum screening and managing cardiometabolic risk factors for the 6% of women diagnosed with GDM each year^1^ could be funded through the annual diabetes and cardiovascular review and provide an opportunity to support effective lifestyle changes and improve pharmaceutical management to reduce adverse outcomes.^12^, ^45^ Based on numbers of people with diabetes on the NZ virtual register,^37^ extending funding for this at‐risk group of women will only increase the total proportion of eligible people in New Zealand with diabetes by 1%–1.5%.

Despite recommendations, there is a paucity of reports on postpartum screening for women diagnosed with GDM internationally,^12^, ^24^ and in New Zealand,^1^, ^44^ with no recommendations for assessment of blood pressure, lipids, smoking status, and physical activity recommendations are minimal.^1^, ^42^ Of the few reports on postpartum screening, most show low rates and poor records for cardiovascular risk factors.^12^, ^24^ Several barriers to screening have been identified, including a lack of knowledge in general practice and guidance on how to systematically screen for type 2 diabetes and communicate the increased risk to women.^6^, ^46^ Despite these limitations, women do prefer to know of their increased risk^47^ and are receptive to prompts for screening, provided they are contextualized for the target population.^46^

Breastfeeding,^45^ lifestyle changes, and metformin for women with GDM, who remain hyperglycemic, are recommended in the postpartum period to reduce the risk of developing type 2 diabetes.^1^, ^42^ A recent meta‐analysis combining 13 lifestyle trials between 4 and 36 months duration computed a risk ratio (0.76 [95% CI: 0.63–0.93]).^48^ A pooled analysis of 16 trials also showed a modest mean weight reduction in these women following delivery (1.8 kg [95% CI: 2.9, 0.6]).^49^ A further challenge for managing and supporting younger women with a history of GDM is the lack of large long‐term lifestyle and pharmaceutical trials, and the difficulty in recruiting mothers with new babies,^50^ to establish a core risk management guideline to reduce adverse outcomes.^51^ Although the NZ guidelines, since 2013, recommend all women diagnosed with GDM are screened for diabetes at 3 months following delivery with an HbA_1c_ and then annually,^1^ postpartum screening is low^1^, ^4^ and significant differences remain between ethnic groups and regions.^44^ Although some centers do routinely recommend annual screening for CVD, current guidelines^1^ require updating to reflect this, and promote evidence‐based lifestyle and pharmaceutical interventions (in addition to metformin) suitable for at‐risk women to improve outcomes.

Main limitations of this study include missing data for some key variables (including body mass index and tobacco use), a lower than ideal positive predictive value and sensitivity using the VDR algorithm,^36^ a high threshold for diagnosis of GDM, a low 50 g polycose sensitivity,^1^ regional variations in screening,^35^ and lower screening rates for Māori women and women residing in regional areas. It is estimated that approximately 4000 women with GDM were not identified in this dataset, accounting for the lower‐than‐expected number of women with GDM. This limitation increases the potential for women with undiagnosed GDM (about 10%) or women not dispensed with antihyperglycemic agents for type 2 diabetes being selected as control women and reduces the effect size. The underestimation of postpartum cardiometabolic outcomes are expected to be exaggerated for Māori and Pacific women. Although there is a paucity of reports for Māori women diagnosed with GDM developing type 2 diabetes, one report showed that >56% of Māori women diagnosed with GDM developed prediabetes or diabetes postdelivery during an average 3.9 year follow‐up and at a mean age of 36 years.^52^ Screening for GDM is notably lower for Māori women,^4^, ^5^, ^6^ and they have increased uptake of the polycose test and are more likely to have a false negative result.^41^ This is because Māori and Pacific women are more likely to be diagnosed with GDM on the fasting 75 g OGTT result.^53^ In New Zealand, we currently have a higher fasting threshold for diagnosis than other countries, so this likely further contributed to the lower than expected rates of GDM in these women.^4^ Postpartum screening rates for Māori and Pacific women are also less than for European and Asian women^44^ and Māori people are less likely to collect dispensed hypoglycemic medication.^54^

The low postpartum smoking prevalence of <5% was far less than the 27% of women with GDM who smoked during pregnancy when initially registered at National Women's Hospital in central Auckland.^4^ The under‐reporting of smoking, which is associated with the development of GDM, type 2 diabetes, renal disease, and CVD^55^ could not be completely controlled for in our analyses and is likely to have resulted in overestimates of HR for each of these outcomes, although our sensitivity analyses suggest this may not have been major.

This report attempts to quantify major long‐term cardiometabolic events in women diagnosed with GDM that are poorly documented and understood.^34^ This large national study of an ethnically diverse population of women with a history of GDM showed a 20‐fold increased risk of developing diabetes, twice the risk of major cardiovascular events, increased mortality, and a five‐fold increase of developing renal disease. Results also quantify the likely contribution of developing cardiovascular and renal disease for women who also developed diabetes following delivery. At‐risk women require annual cardiometabolic reviews to screen for, and effectively manage major cardiovascular risk factors (HbA_1c_, hypertension, dyslipidemia, and smoking cessation) and urinary microalbuminuria. Further research among ethnically diverse groups of women is required to establish effective cardioprotective lifestyle and pharmaceutical interventions for this younger group of at‐risk women.

## AUTHOR CONTRIBUTIONS

Barbara M. Daly and Robert K. R. Scragg conceptualized and designed the study and interpreted the results. Barbara M. Daly acquisitioned the data, conducted aspects of the data analysis, and wrote the manuscript. Robert K. R. Scragg developed the method for the data analyses and contributed to writing the manuscript. Zhenqiang Wu developed the method for the data analyses, analyzed the data, interpreted the results and contributed to writing the manuscript. Krishnarajah Nirantharakumar, Lynne Chepulis, and Janet A. Rowan critically appraised the study findings and reviewed and contributed to the manuscript. Barbara M. Daly, Robert K. R. Scragg, and Zhenqiang Wu had full access to the data and take responsibility for the integrity of the data and the accuracy of the data analysis.

## FUNDING INFORMATION

This study was funded by the University of Auckland 71604/9450.

## CONFLICT OF INTEREST STATEMENT

There are no competing interests relevant to this study.

## Supporting information


**Supplementary File 1** *International Classification of Diseases, Tenth Revision, Australian Modification* (ICD‐10‐AM) codes used for cardiovascular disease events in the Vitamin D Assessment Study and CanterburyHealth Volunteers Study.

## Data Availability

Aggregate data are available on request.
