# Supplementary material for: Increased risk of cardiovascular and renal disease, and diabetes for all women diagnosed with gestational diabetes mellitus in New Zealand—A national retrospective cohort study
Source: J Diabetes. 2024 Apr 10;16(4):e13535. doi: 10.1111/1753-0407.13535 (PMC11006618; doi:10.1111/1753-0407.13535)
Supplement: Supplementary file 1 — International Classification of Diseases, Tenth Revision, Australian Modification (ICD‐10‐AM) codes used for cardiovascular disease events in the Vitamin D Assessment Study and CanterburyHealth Volunteers Study. [file JDB-16-e13535-s001.docx]

**SUPPLEMENTARY FILE**

International Classification of Disease-10-Australian Modification (ICD-10-AM) codes used for cardiovascular disease events in ViDA and HVOLS.

| **Outcome type** | **ICD-10-AM codes** |
| --- | --- |
| Myocardial infarction | **I210, I211 - I214, I219 - I222, I228, I229** |
| Unstable angina | I200 |
| Other coronary heart disease | **I201, I208, I209, I230 - I236, I238, I240, I248, I249, I253 - I256, I460, I469** |
| Ischaemic stroke | **I630 - I636, I638, I639, I64** |
| Haemorrhagic stroke | **I600 - I616, I618, I619** |
| Transient ischaemic attack | G450 - G453, G458 - G468 |
| Peripheral vascular disease | E1050 - E1052, E1150 - E1152, E1451, E1452, I7021 - I7024, I7100 - I7103, I711, I713, I715, I718, I739 - I745, I748, I749 |
| Congestive heart failure | I110, I130, I132, I50, I500, I501, I509 |
| Other ischaemic CVD-related deaths | E1059, E1159, E1459, I250, I2510 - I2513, I252, I258, I259, I461, I650 - I653, I658 - I664, I668 - I670, I672, I690, I691, I693, I694, I698, I700, I701, I7020, I708, I709, I714, Z951, Z955, Z958, Z959. |

* The bolded codes were used to define hard atherosclerotic CVD for external validation of the Pooled Cohort Equations (PCEs). Myocardial infarction and Stroke codes defined fatal and nonfatal events but ‘Other coronary heart disease’ codes were only used to define fatal events in the hard atherosclerotic CVD outcome.
